# Preoperative Circulating Tumor DNA Detection and Risk Stratification in Esophageal Squamous Cell Carcinoma

**DOI:** 10.1001/jamasurg.2025.6755

**Published:** 2026-02-18

**Authors:** Tae Hee Hong, Jun-Gi Jeong, Seong Yong Park, Byung Jo Park, Dongsoo Kyung, Hwang-Phil Kim, Jingjun Zhu, Young Ho Yang, Ha Eun Kim, Chang Young Lee, Jin Gu Lee, Yeong Jeong Jeon, Junghee Lee, Jong Ho Cho, Yong Soo Choi, Young Mog Shim, Se-Hoon Lee, Dae Joon Kim, Hong Kwan Kim

**Affiliations:** 1 Department of Thoracic and Cardiovascular Surgery, Yonsei University College of Medicine, Severance Hospital, Seoul, Korea; 2 Department of Health Sciences and Technology, Samsung Advanced Institute for Health Sciences & Technology, Sungkyunkwan University, Seoul, Korea; 3 Department of Thoracic and Cardiovascular Surgery, Sungkyunkwan University School of Medicine, Samsung Medical Center, Seoul, Korea; 4 IMBdx, Seoul, Republic of Korea; 5 Department of Clinical Research Design and Evaluation, Samsung Advanced Institute for Health Sciences & Technology, Sungkyunkwan University, Seoul, Korea; 6 Division of Hematology-Oncology, Department of Internal Medicine, Sungkyunkwan University, Samsung Medical Center, Seoul, Korea

## Abstract

**Importance:**

Neoadjuvant chemoradiotherapy is recommended for clinical stage II (T2N0) esophageal squamous cell carcinoma (ESCC) with high-risk features such as tumor size 3 cm or greater, lymphovascular invasion (LVI), and poor differentiation. However, these criteria have limited predictive value, and there is an unmet need for more accurate preoperative risk stratification tools.

**Objective:**

To evaluate whether preoperative detection of circulating tumor DNA (ctDNA) is associated with nodal upstaging and postoperative recurrence in patients with clinical stage I (T1b) or T2N0 ESCC.

**Design, Setting, and Participants:**

This cohort study included 2 independent cohorts in Seoul, Korea: Samsung Medical Center (SMC; n = 50), with data from January 2015 through December 2019, and Yonsei University Severance Hospital (YUSH; n = 24), with data from January 2023 through December 2024. All patients had T1b or T2N0 ESCC, underwent radical esophagectomy and lymph node dissection without neoadjuvant therapy, and provided tumor and preoperative plasma samples for tumor-informed ctDNA sequencing.

**Exposure:**

Presence or absence of ctDNA in preoperative plasma samples.

**Main Outcomes and Measures:**

Primary outcomes were pathologic nodal upstaging and survival (recurrence-free survival [RFS] and overall survival [OS]). Associations between ctDNA status and outcomes were analyzed using χ^2^, logistic regression, and Cox proportional hazard models.

**Results:**

In the SMC cohort, the median (IQR) age was 68 (60-74) years, and 47 participants (94%) were male; in the YUSH cohort, the median (IQR) age was 67 (61-69) years, and 21 participants (87.5%) were male. Preoperative ctDNA was detected in 36 patients (48.6%) (27 [54.0%] in SMC and 9 [37.5%] in YUSH). Detection was more frequent in T2N0 than T1b: 26 [57.8%] vs 1 [20.0%] in SMC and 9 [42.9%] vs 0 in YUSH. Over a median (IQR) follow-up of 37.7 (24.9-49.6) months, patients with positive ctDNA results had worse RFS (hazard ratio [HR], 4.15; 95% CI, 1.54-11.22; *P* = .005) and OS (HR, 4.02; 95% CI, 1.50-10.74; *P* = .006). Among patients with T2N0 disease—a subgroup where neoadjuvant therapy decisions remain uncertain—ctDNA positivity predicted occult nodal metastasis with a positive predictive value of 100% (95% CI, 71.51-100) in the SMC cohort and 88.89% (95% CI, 51.75-99.72) in the YUSH cohort. Preoperative ctDNA remained significantly associated with nodal metastasis (odds ratio [OR], 19.98; 3.90-211.42; *P* < .001), outperforming guideline-based criteria (LVI, poor differentiation, tumor size ≥3 cm). Adding ctDNA to models with guideline risk factors improved the area under the receiver operating characteristic curve from 0.66 to 0.91 (*P* = .048) in the SMC cohort, and showed consistent performance in the YUSH cohort (from 0.67 to 0.89; *P* = .03).

**Conclusions and Relevance:**

Preoperative ctDNA detection was significantly associated with occult nodal metastasis and recurrence risk in clinical N0 early-stage ESCC in this study. Among patients with T2N0 ESCC, ctDNA may serve as a complementary biomarker to inform neoadjuvant treatment escalation and support more personalized therapeutic strategies.

## Key Points

**Question:**

Can preoperative circulating tumor DNA (ctDNA) detection help identify high-risk early-stage esophageal squamous cell carcinoma (ESCC), particularly in patients with clinical stage II (T2N0) ESCC where current National Comprehensive Cancer Network guideline risk criteria (≥3 cm, lymphovascular invasion, poor differentiation) are suboptimal?

**Findings:**

In this cohort study, in patients with clinical stage I or II ESCC, preoperative ctDNA positivity was significantly associated with nodal upstaging and poorer survival outcomes. Incorporating ctDNA into guideline-based models improved the prediction of occult lymph node metastasis in patients with T2N0 ESCC, and its predictive performance was reproducible across cohorts.

**Meaning:**

Tumor-informed ctDNA analysis may enhance risk stratification in early-stage ESCC and help inform treatment decisions, and incorporating ctDNA status could aid personalized consideration of neoadjuvant therapy in this challenging subgroup.

## Teaser

This cohort study assesses the use of circulating tumor DNA detection in risk stratification for stage I and II esophageal squamous cell carcinoma.

## Introduction

Curative surgery remains the standard treatment for early-stage esophageal squamous cell carcinoma (ESCC), particularly in patients with clinical stage I or II (T1b and T2N0, respectively) disease.^1,2^ For patients with T2N0 ESCC with high-risk features—such as tumor size 3 cm or greater, lymphovascular invasion (LVI), or poor histologic differentiation—current guidelines recommend neoadjuvant chemoradiotherapy.^2,3^ However, these histopathologic characteristics are typically evaluated using preoperative biopsy specimens, which are prone to sampling error and may underestimate tumor aggressiveness due to intratumoral heterogeneity.^4^ In addition, radiologic nodal staging often fails to detect microscopic metastases, resulting in unexpected nodal upstaging after surgery.^5,6^ These limitations highlight the need for more accurate and reproducible biomarkers to improve preoperative risk stratification in early-stage ESCC.^7^

Circulating tumor DNA (ctDNA) has emerged as a promising biomarker for prognostication and molecular residual disease monitoring in solid tumors.^8,9,10^ When detected preoperatively, ctDNA may reflect tumor burden and underlying biological aggressiveness, even in radiographically localized disease. In early-stage non–small cell lung cancer, pretreatment ctDNA positivity has been associated with increased risk of recurrence and occult nodal involvement, suggesting its utility in guiding treatment escalation.^11,12^ Despite growing interest in ctDNA, its role in early-stage ESCC remains largely unexplored, particularly with respect to its ability to identify patients with clinically occult nodal metastases.

In this study, we evaluated preoperative ctDNA using a tumor-informed, targeted deep sequencing approach in patients with T1b or T2N0 ESCC who underwent curative surgery without neoadjuvant therapy. We assessed whether ctDNA positivity was associated with pathologic nodal upstaging and postoperative survival outcomes. Additionally, we examined whether incorporating ctDNA into existing guideline-based models could improve the prediction of occult lymph node metastasis. Our aim was to determine the potential of ctDNA as a complementary biomarker for refining preoperative risk assessment and optimizing treatment strategies in early-stage ESCC.

## Methods

### Study Participants

This retrospective study included 2 independent cohorts of patients with T1b or T2N0 ESCC who underwent curative resection without neoadjuvant therapy. For this multi-institutional analysis, the primary cohort comprised 50 patients from Samsung Medical Center (SMC; Seoul, Korea) diagnosed with clinical stage T1b to T3N0M0 ESCC between January 2015 and December 2019, and 24 patients from Yonsei University Severance Hospital (YUSH; Seoul, Korea) diagnosed with clinical stage T1b to T2N0M0 ESCC between January 2023 and December 2024. All patients had biopsy-confirmed ESCC, available formalin-fixed paraffin-embedded tumor samples, and preoperative plasma collected within 2 weeks before surgery. Patients with a prior malignancy within 5 years, receipt of neoadjuvant therapy, or insufficient biospecimen availability were excluded, as detailed in eFigure 1A in Supplement 1.

Clinicopathologic and staging data, as well as preoperative blood and tumor specimens, were prospectively captured under institutional review board–approved biobank registries at both centers as part of ongoing precision-oncology initiatives, with standardized electronic case-report forms monitored by independent data managers. Both institutions applied identical eligibility criteria, though practice differences led to inclusion of select cT3N0 cases at SMC but not at YUSH. All participants provided written informed consent, and the study was approved by both institutional review boards. This study followed the Strengthening the Reporting of Observational Studies in Epidemiology (STROBE) reporting guideline.

### Preoperative Evaluation, Surgical Approach, and Follow-Up Protocol

All patients at both institutions underwent comprehensive preoperative staging, including contrast-enhanced chest and abdominal computed tomography, positron emission tomography–computed tomography, and endoscopic ultrasonography. All patients at both institutions underwent radical esophagectomy with standard 2-field or 3-field lymphadenectomy, depending on tumor location and clinical stage. Minimally invasive esophagectomy—using video-assisted thoracoscopic surgery or robotic-assisted thoracoscopic surgery—was the preferred approach at both centers whenever technically feasible. The choice between Ivor Lewis and McKeown procedures was determined based on tumor location, stage, and surgeon discretion. Gastric conduit reconstruction was performed in all cases. Lymphadenectomy followed institutional protocols consistent with either the National Comprehensive Cancer Network (NCCN) guidelines or the Japanese Esophageal Society standards to ensure adequate nodal harvest for pathologic staging.^2,13^ In the SMC cohort, patients underwent regular follow-up with physical examinations, blood tests, and chest computed tomography or positron emission tomography–computed tomography every 3 months for 2 years, then every 6 months. Outcomes for those lost to follow-up were assessed by phone. In the YUSH cohort, patients had chest and abdominopelvic computed tomography scans every 4 to 6 months for 2 years, then every 6 months. Locoregional recurrence was defined as recurrence in the surgical field or anastomosis site; distant recurrence as metastasis to distant organs or nonregional nodes. Recurrence was diagnosed clinically and confirmed by positron emission tomography or biopsy.

### Pathologic Assessment

Preoperative endoscopic biopsy specimens were used to assess tumor grade (1 to 3) and the presence of LVI. Tumor length was documented based on endoscopic examination reports. After surgical resection, pathologic staging was performed according to the 8th edition of the American Joint Committee on Cancer tumor/node/metastasis (TNM) classification. Each specimen was evaluated for tumor depth, nodal status, and distant metastasis when applicable.

Additional histopathologic features—including LVI, perineural invasion, nuclear grade, and resection margin status—were assessed as part of routine diagnostic practice. All evaluations were performed by board-certified pathologists at each institution in accordance with institutional protocols and international pathology standards.

### Cell-Free DNA Sequencing and Interpretation

Preoperative plasma samples, submitted within 2 weeks prior to surgery, were analyzed using the AlphaLiquid 100 assay (IMBdx), a clinically validated, tumor-informed targeted cell-free DNA (cfDNA) sequencing panel covering 118 cancer-associated genes.^14^ cfDNA libraries incorporated unique molecular identifiers and error-suppression technology to enhance analytical sensitivity and specificity. Sequencing was performed at a target depth of median deduplicated depth of approximately 50 000 to enable high-sensitivity detection of somatic mutations and copy number variations. Samples with insufficient input cfDNA were excluded according to the manufacturer’s recommendations (eMethods in Supplement 1). Matched tumor tissue genomic DNA was extracted and sequenced using the AlphaSolid assay, which targets the same 118 genes as AlphaLiquid. Tumor sequencing was conducted at a target depth of 500.

ctDNA positivity was defined as the presence of plasma variants that matched somatic variants identified in the corresponding tumor tissue. A variant allele frequency threshold of 0.1% was used as the limit of detection; variants below this threshold were considered unreliable due to insufficient supporting read counts. For *TP53*, a higher variant allele frequency cutoff of 0.2% was applied due to its known susceptibility to clonal hematopoiesis-associated alterations.^15^ All variants were curated according to the joint Association for Molecular Pathology, the American Society of Clinical Oncology, and the College of American Pathologists guidelines for somatic variant interpretation.^16^ Additional validation of the variant allele frequency threshold and cfDNA input quality is described in the eMethods and eFigure 2 in Supplement 1. In this study, the AlphaLiquid 100 assay was used for research purposes under institutional review board–approved protocols, and ctDNA detection results did not influence clinical management or treatment decisions.

### Outcomes

The primary end point was to assess the predictive performance of preoperative ctDNA detection for occult nodal metastasis (nodal upstaging). Nodal upstaging analysis was specifically performed in the T2N0 subgroup, a clinically important category where treatment decisions regarding neoadjuvant therapy remain controversial. Secondary end points included recurrence-free survival (RFS) and overall survival (OS), defined as the time from surgery to recurrence, death, or last follow-up. Survival analyses were conducted in the SMC cohort using Kaplan-Meier estimates and Cox proportional hazards models. As potential covariates in the Cox proportional hazard model, age, clinical T stage, tumor size, LVI, and tumor grade were initially evaluated in univariable analyses. Clinical N stage was not included because all patients were clinically node-negative at baseline. Variables showing *P* < .20 or recognized as established risk factors in the NCCN Clinical Practice Guidelines or American Joint Committee on Cancer 8th edition were subsequently included in the multivariable Cox model. The proportional hazards assumption was evaluated using Schoenfeld residuals and visual inspection of residual plots. Minor deviations for ctDNA were addressed by performing sensitivity analyses with restricted follow-up durations, which confirmed model stability and consistent hazard ratio estimates.

### Statistical Analysis

Associations between ctDNA status and clinicopathologic variables were examined using χ^2^ tests and logistic regression. The predictive value of ctDNA for occult nodal metastasis was assessed using receiver operating characteristic (ROC) curves and area under the curve (AUC), initially in the SMC cohort and subsequently validated in the YUSH cohort. AUC comparisons were used to evaluate the added value of ctDNA beyond NCCN guideline–based risk factors (tumor size ≥3 cm, poor differentiation, and LVI). Independence between ctDNA detection and these guideline-based risk factors was evaluated using Fisher exact test. Model calibration was evaluated using the Hosmer-Lemeshow test (5 risk groups) and calibration plots displaying predicted vs observed probabilities with 95% CIs, as described in eFigure 3 in Supplement 1. Net reclassification index, integrated discrimination improvement, and bootstrap validation were performed to assess model improvement when ctDNA was added to conventional risk factors. Risk categories were defined as low (<0.3), intermediate (0.3-0.7), and high (≥0.7) based on predicted probabilities. All statistical analyses were performed using R version 4.3.2 (R Foundation), including the PredictABEL and nricens packages for reclassification analyses, with 2-sided *P* values <.05 considered statistically significant.

## Results

### Patient Characteristics

A total of 50 patients with T1b or T2N0 ESCC from SMC and 24 patients from YUSH were included in the analysis (eFigure 1B in Supplement 1). All patients underwent curative esophagectomy without neoadjuvant therapy and had paired tumor and preoperative plasma samples available for ctDNA analysis. Baseline demographic and clinical characteristics are summarized in eTable 1 in Supplement 1.

In the SMC cohort, the median (IQR) age was 68 (60-74) years; 47 participants (94%) were male and 3 (6%) were female. In the YUSH cohort, the median (IQR) age was 67 (61-69) years; 21 participants (87.5%) were male and 3 (12.5%) were female. Clinical T2 tumors accounted for 48% of cases (n = 24), and all patients were clinically node negative. The YUSH cohort showed similar distributions. Preoperative ctDNA was detected in 27 participants (54.0%) in SMC and 9 (37.5%) in YUSH. Detection rates were comparable across cohorts, allowing for consistent downstream analysis.

### Genomic Profiles From Tumor Tissue and Plasma

**Figure 1.**
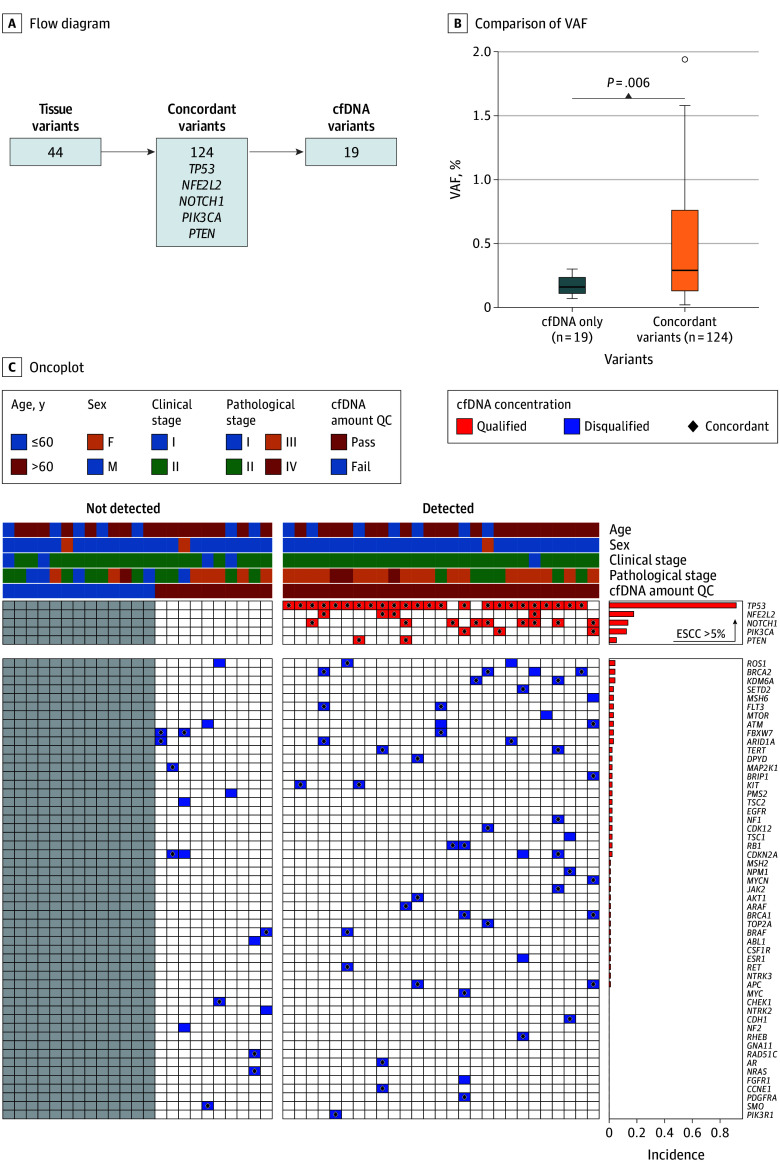
Genomic Profiling and Clinical Characteristics Stratified by Circulating Tumor DNA (ctDNA) Detection Status in Patients With Esophageal Squamous Cell Carcinoma (ESCC) A, Flow diagram depicting variant distribution between tissue and cfDNA samples. B, Comparison of variant allele frequencies (VAF) between cell-free DNA (cfDNA)–only variants (n = 19) and concordant variants (n = 124). C, Oncoplot displaying clinical characteristics and genomic alterations in patients with not-detected vs detected ctDNA status. QC indicates quality control.

Genomic profiling identified 187 somatic variants across all tumor and plasma samples. Of these, 124 variants (66.3%) were concordant between matched tumor tissue and plasma samples, while 44 variants (23.5%) were detected exclusively in tumor tissue and 19 (10.2%) were found only in plasma (Figure 1A). Notably, key cancer-associated genes, such as *TP53*, *NFE2L2*, *NOTCH1*, *PIK3CA*, and *PTEN*, were frequently observed among concordant variants. These findings reflect the representative genomic landscape of ESCC.

Variant allele frequencies of concordant variants were significantly higher than those of cfDNA-only variants (median [IQR], 0.29% [0.13-0.76] vs 0.16% [0.11-0.24]; *P* = .006), supporting their analytical reliability (Figure 1B). We also stratified genomic alterations by ctDNA detection status (Figure 1C). Canonical ESCC genes (occurring at >5% frequency in TCGA ESCC cohort) was more frequently detected in the ctDNA-positive group, further validating the relevance of our tumor-informed ctDNA detection strategy.

### Association Between ctDNA Detection and Tumor Burden

**Figure 2.**
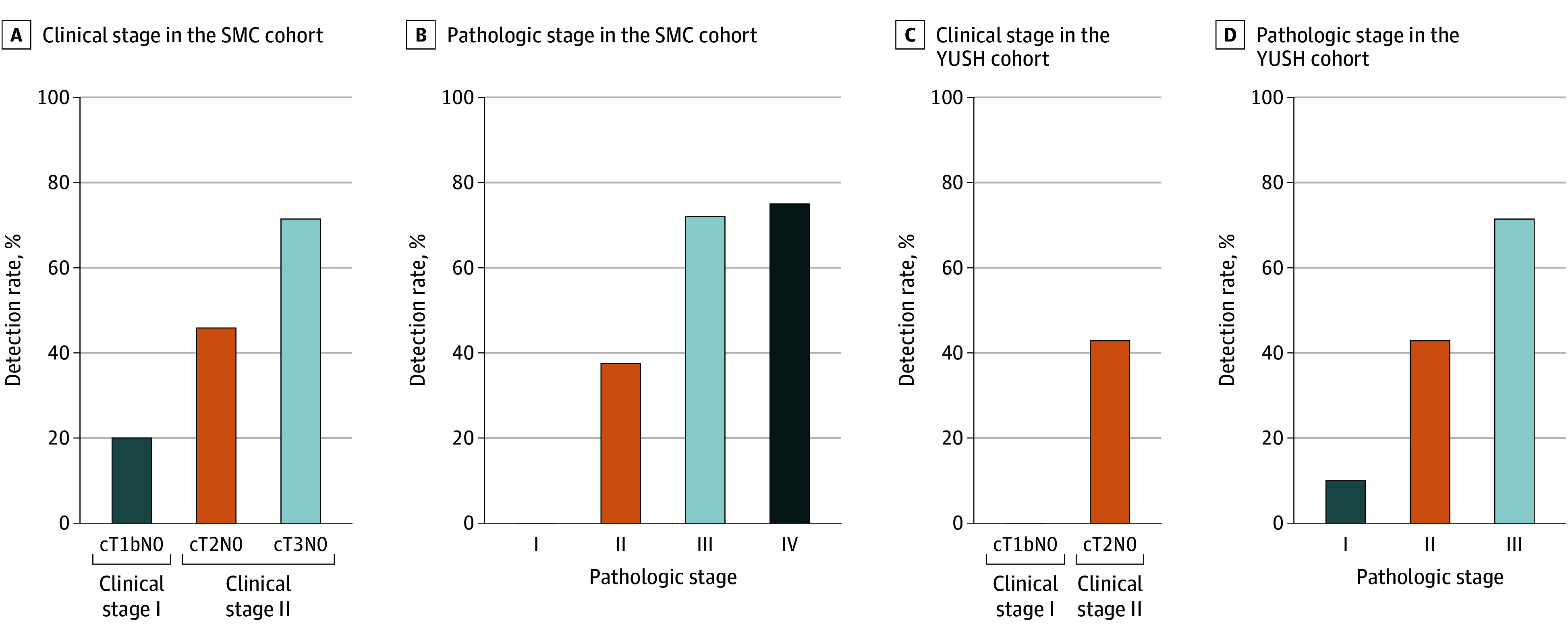
Circulating Tumor DNA (ctDNA) Detection Rates by Clinical and Pathologic Stage in Patients With Esophageal Squamous Cell Carcinoma (ESCC) A, Detection rate of ctDNA stratified by clinical stage in the Samsung Medical Center (SMC) cohort (n = 50). B, Detection rate of ctDNA according to pathologic stage in the SMC cohort. C, Detection rate of ctDNA by clinical stage in the Yonsei University Severance Hospital (YUSH) cohort (n = 24). D, Detection rate of ctDNA by pathologic stage in the YUSH cohort.

Across both cohorts, ctDNA positivity was associated with greater tumor extent. In the SMC cohort, ctDNA was detected in 1 of 5 patients with T1bN0 ESCC (20.0%), 11 of 24 with T2N0 (45.8%), and 15 of 21 with cT3N0 (71.4%) (Figure 2A). Similarly, in the YUSH cohort, detection was 0 of 3 (0.0%) in patients with T1bN0 ESCC and 9 of 21 with T2N0 (42.9%) (Figure 2C).

In the SMC cohort, ctDNA detection increased progressively with pathologic stage: 0 of 5 (0.0%) in pathological stage I, 6 of 16 (37.5%) in pathological stage II, 18 of 25 (72.0%) in pathological stage III, and 3 of 4 (75.0%) in pathological stage IV (Figure 2B). In the YUSH cohort, ctDNA was observed in 1 of 10 (10.0%) pathological stage I, 3 of 7 (42.9%) pathological stage II, and 5 of 7 (71.4%) pathological stage III patients (Figure 2D). These findings support the notion that ctDNA positivity correlates with clinical and pathological tumor burden across disease stages. Corresponding Sankey diagrams illustrate concordance between clinical and pathological stages by ctDNA detection status in both cohorts (eFigure 4A and 4B in Supplement 1).

### Prognostic Impact of Preoperative ctDNA and Recurrence Pattern

**Figure 3.**
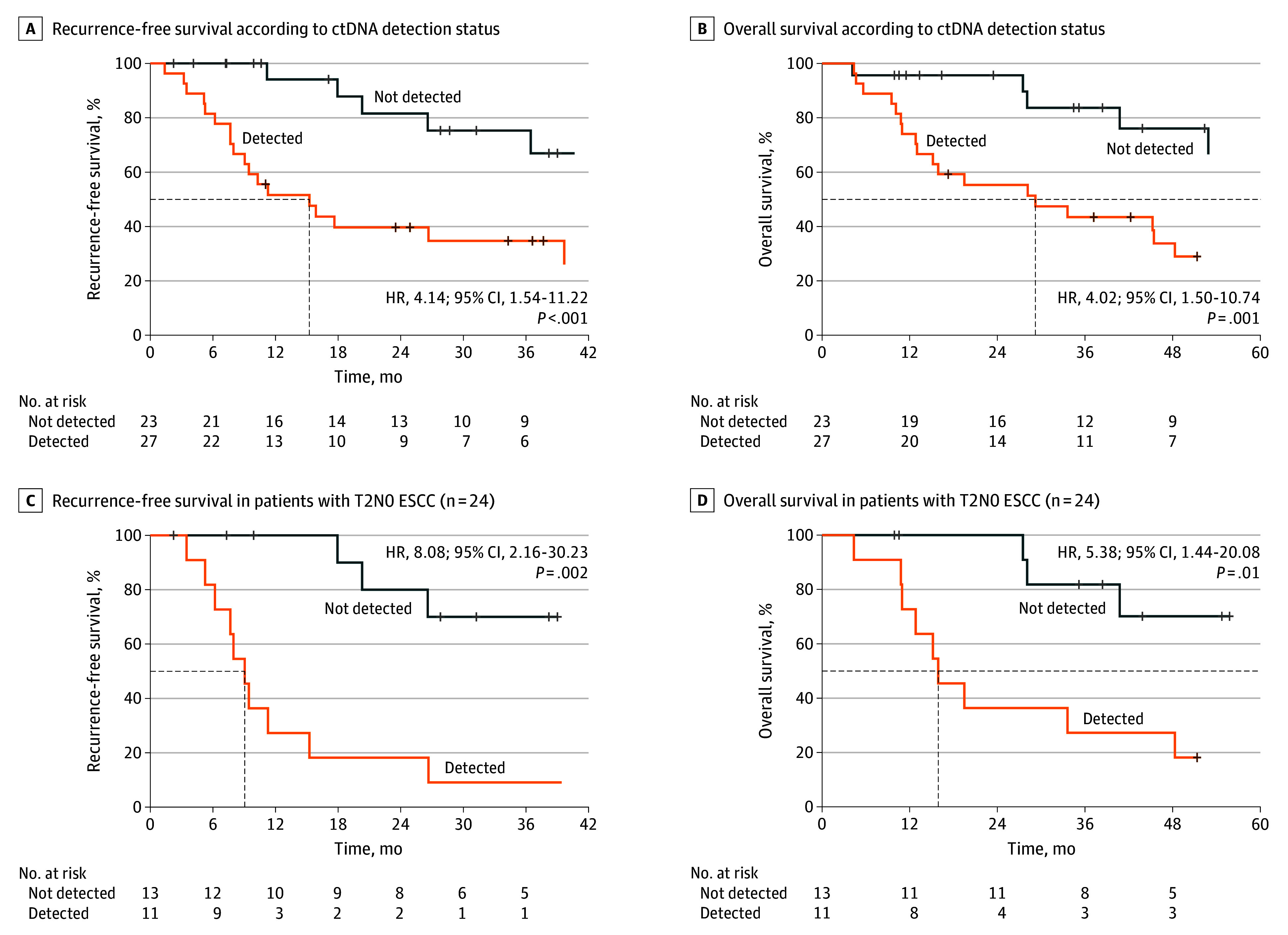
Prognostic Impact of Preoperative Circulating Tumor DNA (ctDNA) Status in Patients With Esophageal Squamous Cell Carcinoma (ESCC) HR indicates hazard ratio; T2N0, clinical stage II.

The median follow-up duration in the SMC cohort was 53.5 months (95% CI, 43.9-61.8) (eFigure 5A and 5B in Supplement 1). Preoperative ctDNA positivity was significantly associated with worse RFS (hazard ratio [HR], 4.15; 95% CI, 1.54-11.22; *P* = .005) and OS (HR, 4.02; 95% CI, 1.50-10.74; *P* = .006), compared to ctDNA-negative patients (Figure 3A and B). In the T2N0 subgroup, similar trends were observed. Patients with positive ctDNA results had significantly inferior RFS (HR, 8.08; 95% CI, 2.16-30.23; *P* = .002) and OS (HR, 5.38; 95% CI, 1.44-20.08; *P* = .01) compared to those with negative ctDNA results (Figure 3C and D).

In multivariable Cox regression analysis adjusting for age, clinical T and N stage, preoperative tumor size, LVI, and tumor grade, ctDNA positivity remained independently associated with both RFS (adjusted HR, 4.07; 95% CI, 1.27-13.01; *P* = .02) and OS (adjusted HR, 3.54; 95% CI, 1.24-10.05; *P* = .02) (eTable 2 and eTable 3 in Supplement 1). In the sensitivity analyses using reduced 3- and 4-variable models, ctDNA remained significantly associated with RFS (HR, 3.47; 95% CI, 1.26-9.53; *P* = .02 and HR, 3.86; 95% CI, 1.31-11.39; *P* = .02, respectively).

eTable 4 in Supplement 1 further shows that patients with positive ctDNA results exhibited significantly more advanced pathologic T and N stages—including higher rates of pT3 tumors (69.4% vs 26.3%; *P* < .001) and pN2-3 nodal involvement (52.8% vs 10.5%; *P* < .001). This highlights the potential of ctDNA to uncover biologically aggressive tumors with occult progression that are not detected by conventional clinical staging.

Regarding recurrence patterns, ctDNA-positive patients experienced a higher proportion of systemic recurrences compared to patients with negative ctDNA results (48.1% vs 13.0%; *P* = .01), while there was no significant difference in locoregional recurrence rates between the 2 groups (18.5% vs 8.7%; *P* = .43) (eTable 5 in Supplement 1).

### ctDNA Detection and Occult Lymph Node Metastasis

**Figure 4.**
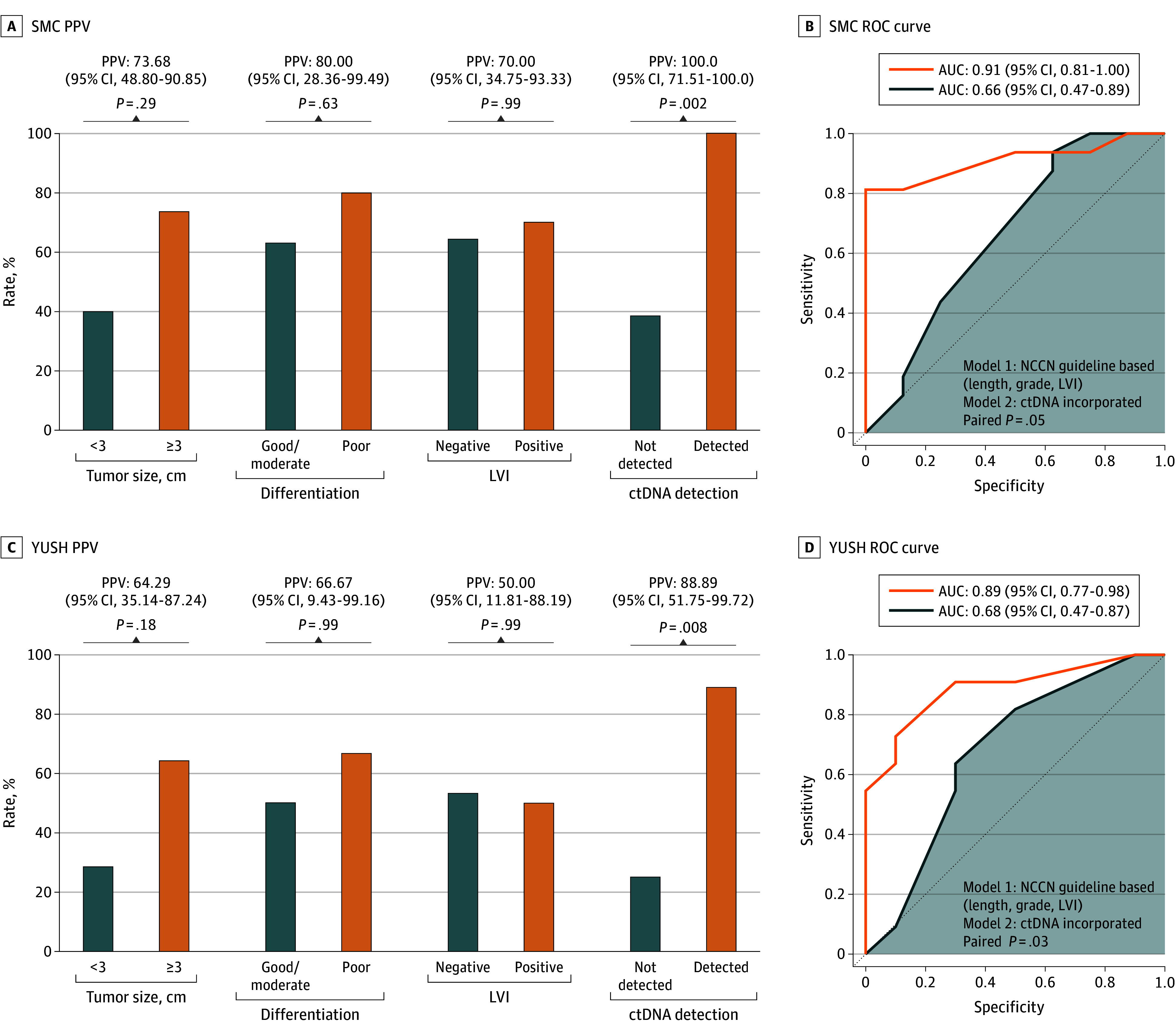
Predictive Value of Circulating Tumor DNA (ctDNA) Status for Occult Nodal Metastasis in Patients With Clinical Stage II Esophageal Squamous Cell Carcinoma (ESCC) A, Positive predictive values (PPVs) of conventional high-risk factors and ctDNA detection for pathologic nodal metastasis in the Samsung Medical Center (SMC) cohort. B, Receiver operating characteristic curves showing the added value of ctDNA incorporation to guideline-based risk assessment for predicting nodal metastasis in the SMC cohort. C, Predictive performance of ctDNA and conventional risk factors in the Yonsei University Severance Hospital (YUSH) cohort. D, Receiver operating characteristic curves showing the added value of ctDNA incorporation to guideline-based risk assessment for predicting nodal metastasis in the YUSH cohort. LVI indicates lymphovascular invasion.

Given the strong prognostic association, we next examined the predictive value of ctDNA for occult nodal metastasis, particularly in the clinical T2N0 subgroup. Among 45 patients with T2N0 from both institutions with complete nodal staging data, pathologic nodal metastasis was identified in 27 (60%). In the pooled analysis, ctDNA detection demonstrated exceptional positive predictive value for occult nodal metastasis, with 19 of 20 patients with positive ctDNA results (95.00%; 95% CI, 75.13-99.87) harboring pathologic nodal disease (eFigure 6A in Supplement 1). This high positive predictive value was observed with overall diagnostic metrics of sensitivity 70.37% (95% CI, 49.82-86.25), specificity 94.44% (95% CI, 72.71-99.86), and negative predictive value 68.00% (95% CI, 46.50-85.05) (eTable 6 in Supplement 1). In the SMC cohort, all patients with positive ctDNA results (11/11) had pathologic nodal metastasis, yielding a positive predictive value (PPV) of 100% (95% CI, 71.51-100) (Figure 4A). In the YUSH cohort, nodal metastasis was found in 8 of 9 ctDNA-positive patients (PPV, 88.89%; 95% CI, 51.75-99.72) (Figure 4C).

In multivariable logistic regression, ctDNA detection remained significantly associated with nodal metastasis (odds ration [OR], 19.98; 95% CI, 3.90-211.42; *P* < .001), even after adjusting for tumor size, histologic grade, and LVI (Table). This association remained significant in stratified multivariable analyses performed separately within each cohort, confirming the robustness of ctDNA as a predictor of nodal involvement across both cohorts (eTable 7 and eTable 8 in Supplement 1). To better quantify the clinical value of ctDNA testing across early-stage disease, we conducted a pooled analysis of all patients with cT1 to 2N0 ESCC (n = 53), in which ctDNA remained a strong independent predictor of occult nodal metastasis (OR, 19.11; 95% CI, 4.01-189.44; *P* < .001) with a positive predictive value of 95.0% (95% CI, 75.1-99.9). These findings support the consistent utility of ctDNA in identifying high-risk patients across both T1b and T2N0 ESCC (eTable 9 in Supplement 1).

**Table.** Logistic Regression Analysis for Prediction of Nodal Metastasis in Patients With Clinical Stage II Esophageal Squamous Cell Carcinoma (ESCC)

Characteristic	Univariable		Multivariable	
	OR (95% CI)	*P* value	OR (95% CI)	*P* value
Tumor size^a^				
<3 cm	NA	NA	NA	NA
≥3 cm	3.49 (0.95-14.01)	.07	1.75 (0.40-8.30)	.46
Grade^a^				
1-2	NA	NA	NA	NA
3	1.39 (0.32-7.36)	.67	0.82 (0.11-5.17)	.83
LVI^a^				
Negative	NA	NA	NA	NA
Positive	1.14 (0.34-4.08)	.83	0.50 (0.08-2.33)	.39
ctDNA				
Not detected	NA	NA	NA	NA
Detected	22.90 (4.71-228.24)	<.001	19.98 (3.90-211.42)	<.001

Abbreviations: OR, odds ratio; ctDNA, circulating-tumor DNA; LVI, lymphovascular invasion.

^a^ Multivariable model included tumor size, ctDNA status, grade, and LVI based on clinical relevance and guideline recommendations. Tumor grade was defined based on the degree of histologic differentiation according to the World Health Organization classification: grade 1 (well differentiated), grade 2 (moderately differentiated), and grade 3 (poorly differentiated).

Incorporating ctDNA status into a model based on NCCN guideline–defined high-risk features significantly improved the AUC from 0.66 (95% CI, 0.47-0.81) to 0.87 (95% CI, 0.75-0.96; *P* < .001) (eFigure 6B in Supplement 1). In individual institutional analyses, AUC improvement was observed from 0.66 (95% CI, 0.47-0.89) to 0.91 (95% CI, 0.81-1.00; *P* = .048) in the SMC cohort (Figure 4B), and from 0.68 (95% CI, 0.47-0.87) to 0.89 (95% CI, 0.77-0.98; *P* = .03) in the YUSH cohort (Figure 4D), demonstrating the incremental utility of ctDNA in predicting nodal upstaging beyond conventional clinical criteria. The ctDNA-incorporated model showed ideal calibration (slope, 1.0; Brier scores, 0.11-0.13) across institutional cohorts (eTable 10 in Supplement 1). Net reclassification analysis confirmed substantial improvement in risk classification when ctDNA was incorporated (categorical net reclassification index, 1.11; 95% CI, 0.77-1.45; *P* < .001) (eTable 11 in Supplement 1).

## Discussion

In this cohort study, we demonstrated that preoperative detection of ctDNA in patients with T1b or T2N0 ESCC was associated with occult nodal metastasis discovered after surgery and remained significantly associated with inferior RFS and OS. These findings position ctDNA as a promising biomarker for improving preoperative risk stratification in early-stage ESCC, particularly in guiding decisions regarding treatment escalation with neoadjuvant therapy.

Over the past decade, accumulating evidence has supported the prognostic value of ctDNA in esophageal cancer.^17,18,19,20,21^ In esophageal adenocarcinoma, ctDNA positivity prior to surgery has been linked to increased tumor burden, including locoregionally advanced tumors, nodal involvement, and distant metastasis.^19,20^ Similarly, in cohorts of ESCC patients receiving neoadjuvant therapy, preoperative ctDNA positivity has been associated with significantly lower 3-year RFS compared to that in patients with negative ctDNA results, and ultradeep sequencing studies have identified ctDNA as an independent predictor of recurrence and mortality.^21^ However, most existing studies have focused on locally advanced or posttreatment settings, and few have specifically addressed the clinical utility of ctDNA in early-stage (T1b or T2N0) ESCC, which is the predominant subtype in East Asia. This unmet need prompted our investigation into whether ctDNA could help identify high-risk patients within the T1b and T2N0 subgroup prior to upfront surgery.

Occult nodal metastasis is not uncommon even in patients with early-stage ESCC, with reported rates of approximately 30% to 40% in T1b and up to 50% in cT2 tumors.^5,6^ Its presence is a major determinant of postoperative prognosis, yet accurate preoperative identification remains challenging. Current treatment guidelines recommend neoadjuvant chemoradiotherapy for T2N0 tumors only when additional high-risk features—such as tumor size 3 cm or greater, poor differentiation, or LVI—are suspected^2,3^; however, these pathologic factors are frequently underestimated due to the inherent limitations of preoperative biopsy and imaging. This diagnostic gap underscores the need for more reliable biomarkers. Our findings highlight ctDNA as a promising noninvasive tool to detect biologically aggressive disease and select candidates who may benefit from neoadjuvant chemoradiotherapy, even among patients classified as early-stage by conventional clinical criteria.

Notably, ctDNA positivity demonstrated exceptionally high positive predictive value for pathologic nodal metastasis—100% in the SMC cohort and 88.89% in the YUSH cohort. When incorporated into multivariable prediction models, ctDNA significantly enhanced the accuracy of nodal upstaging prediction beyond NCCN guideline-based risk factors. Furthermore, patients with positive ctDNA results experienced markedly worse postoperative outcomes, reinforcing the clinical relevance of ctDNA as both a predictive and prognostic tool in early-stage ESCC.

In our cohort, patients with positive ctDNA results showed predominantly distant rather than locoregional relapse, suggesting that preoperative ctDNA reflects micrometastatic systemic disease rather than residual local tumor burden. This observation supports consideration of systemic control-oriented perioperative strategies. Recent trials, including JCOG1109 (NeXT),^22^ demonstrated improved survival with triplet chemotherapy compared with conventional chemotherapy, although its impact on systemic relapse reduction remains uncertain. In contrast, early-phase chemoimmunotherapy studies have shown encouraging response rates, raising the possibility of enhanced systemic disease control.^23^ Collectively, these findings highlight the need for prospective ctDNA-guided studies to determine optimal systemic regimens for ctDNA-positive ESCC.

Although some patients with negative ctDNA results were later found to have nodal metastases, most exhibited only limited pN1 disease, and all underwent complete surgical resection with lymphadenectomy, ensuring accurate staging and appropriate adjuvant treatment. In our T2N0 subgroup, the negative predictive value was 61.5%, and only 2 patients had more extensive pN2-3 involvement. While the possibility of false negatives highlights the need for assay sensitivity optimization, the current treatment paradigm—with upfront surgery for patients with negative ctDNA results—remains clinically safe and oncologically sound. This balance supports the role of ctDNA in identifying high-risk patients without compromising outcomes for those with negative test results.

Our findings support the need for prospective validation of ctDNA-guided treatment escalation strategies in high-risk, early-stage ESCC. In particular, patients with clinical stage T1b or T2N0 ESCC represent a therapeutic gray zone where the role of neoadjuvant therapy remains controversial. A molecularly guided approach using ctDNA may help refine treatment selection in this subgroup. Future clinical trials evaluating ctDNA-informed neoadjuvant chemoradiotherapy in patients with T1b or T2N0 ESCC and positive ctDNA results are warranted and may lay the groundwork for more personalized and risk-adapted management algorithms in early-stage ESCC.

### Limitations

Several limitations of this study should be acknowledged. First, the sample size was modest (particularly n = 45 in the T2N0 subgroup) and limited to 2 Korean academic institutions, and without separate development and validation cohorts, which may affect the generalizability of the findings and raise the possibility of overfitting. The wide confidence intervals for some risk estimates and the absence of predefined clinical thresholds or a standardized risk scoring system limit immediate clinical applicability. Second, although all patients underwent curative-intent esophagectomy with radical lymphadenectomy, staging practices, surgical decision-making, and biobank workflows differed across institutions, leading to variation in exclusion rates and contributing to sample-size constraints. These institutional differences, together with heterogenous event distributions, likely contributed to the modest calibration discrepancies observed in the pooled analysis. Third, to better reflect real-world preoperative workflows, ctDNA testing ideally requires somatic variant data derived from endoscopic biopsy specimens. However, due to the limited availability of adequately preserved endoscopic samples, surgical specimens were used for tumor-informed sequencing in this study. Fourth, our assay was tumor informed but not personalized, relying on a fixed panel, and thus has lower sensitivity—especially in low-burden tumors. Personalized assays like Signatera may offer higher sensitivity via deep sequencing of patient-specific variants but require longer turnaround times. These trade-offs highlight the need for further optimization in the preoperative setting.

## Conclusions

In conclusion, this is, to our knowledge, the first study to evaluate the association between pretreatment ctDNA status and postsurgical pathologic and prognostic outcomes in T1b or T2N0 ESCC. Preoperative ctDNA detection was associated with occult nodal metastasis and poor survival, suggesting its utility in refining treatment selection—particularly in patients with T2N0 ESCC where conventional preoperative risk factors are insufficient. Prospective validation is needed to establish ctDNA as a clinical decision-making tool and to determine its role in guiding neoadjuvant therapy in early-stage ESCC.

## Notes

Supplement 1. eMethods eFigure 1. Study workflow and patient enrollment across institutional cohorts. (A) CONSORT-style diagram depicting patient screening, exclusion, and inclusion for both institutional cohorts (SMC and YUSH). (B) Schematic representation of the study workflow eFigure 2. Validity of cfDNA analytical parameters. (A) Alteration depth stratified by cfDNA VAF threshold of 0.1%. (B) cfDNA sequencing depth according to cfDNA input quantity (≤cutoff vs. >cutoff). (C) cfDNA amount (ng) compared between pathologic stage I and stage II–IV eFigure 3. Model calibration plots for prediction of nodal metastasis in clinical T2N0 ESCC patients. (A) Calibration plot for the SMC cohort (n=24). (B) Calibration plot for the YUSH cohort (n=21). (C) Calibration plot for the pooled cohort (n=45).eTable 1. Clinicopathological characteristics of study population eTable 1. Clinicopathological characteristics of study population eFigure 4. Association between clinical stage, ctDNA detection status, and pathologic stage in ESCC patients. (A) Sankey diagram displaying the relationship between clinical stage, ctDNA detection status, and pathologic stage in the SMC cohort. (B) Sankey diagram displaying the relationship between clinical stage, ctDNA detection status, and pathologic stage in the YUSH cohort eFigure 5. Survival outcomes in the overall ESCC cohort. (A) Kaplan-Meier curve for recurrence-free survival of all patients. (B) Kaplan-Meier curve for overall survival of all patients eTable 2. Univariable and multivariable Cox regression analysis using preoperative risk variables for recurrence-free survival eTable 3. Univariable and multivariable Cox regression analysis using preoperative risk variables for overall survival eTable 4. Pathologic stage distribution by ctDNA status eTable 5. Association between preoperative ctDNA detection and recurrence patterns eFigure 6. Predictive performance of ctDNA for occult nodal metastasis in the pooled cT2N0 cohort. (A) Positive predictive values (PPV) of conventional high-risk factors and ctDNA detection for pathologic nodal metastasis in the pooled cT2N0 cohort. (B) Receiver operating characteristic curves showing the added value of ctDNA incorporation to guideline-based risk assessment for predicting nodal metastasis in the pooled cT2N0 cohort eTable 6. Diagnostic Performance Metrics of ctDNA and Conventional Risk Factors for Predicting Occult Nodal Metastasis in Clinical T2N0 ESCC Patients eTable 7. Logistic regression analysis for prediction of nodal metastasis in the SMC cT2N0 cohort (N=24) eTable 8. Logistic regression analysis for prediction of nodal metastasis in the YUSH cT2N0 cohort (N=21) eTable 9. Logistic regression analysis for prediction of nodal metastasis in the combined cT1-2N0 cohort (N=53) eTable 10. Model Calibration and Discrimination Metrics for Prediction of Nodal Metastasis eTable 11. Net Reclassification Index Analysis

Supplement 2. Data sharing statement
