# Supplementary material for: Preoperative Circulating Tumor DNA Detection and Risk Stratification in Esophageal Squamous Cell Carcinoma
Source: JAMA Surg. 2026 Feb 18;161(4):370–9. doi: 10.1001/jamasurg.2025.6755 (PMC12917745; doi:10.1001/jamasurg.2025.6755)
Supplement: Supplement 1. — eMethods eFigure 1. Study workflow and patient enrollment across institutional cohorts. (A) CONSORT-style diagram depicting patient screening, exclusion, and inclusion for both institutional cohorts (SMC and YUSH). (B) Schematic representation of the study workflow eFigure 2. Validity of cfDNA analytical parameters. (A) Alteration depth stratified by cfDNA VAF threshold of 0.1%. (B) cfDNA sequencing depth according to cfDNA input quantity (≤cutoff vs. >cutoff). (C) cfDNA amount (ng) compared between pathologic stage I and stage II–IV eFigure 3. Model calibration plots for prediction of nodal metastasis in clinical T2N0 ESCC patients. (A) Calibration plot for the SMC cohort (n=24). (B) Calibration plot for the YUSH cohort (n=21). (C) Calibration plot for the pooled cohort (n=45).eTable 1. Clinicopathological characteristics of study population eTable 1. Clinicopathological characteristics of study population eFigure 4. Association between clinical stage, ctDNA detection status, and pathologic stage in ESCC patients. (A) Sankey diagram displaying the relationship between clinical stage, ctDNA detection status, and pathologic stage in the SMC cohort. (B) Sankey diagram displaying the relationship between clinical stage, ctDNA detection status, and pathologic stage in the YUSH cohort eFigure 5. Survival outcomes in the overall ESCC cohort. (A) Kaplan-Meier curve for recurrence-free survival of all patients. (B) Kaplan-Meier curve for overall survival of all patients eTable 2. Univariable and multivariable Cox regression analysis using preoperative risk variables for recurrence-free survival eTable 3. Univariable and multivariable Cox regression analysis using preoperative risk variables for overall survival eTable 4. Pathologic stage distribution by ctDNA status eTable 5. Association between preoperative ctDNA detection and recurrence patterns eFigure 6. Predictive performance of ctDNA for occult nodal metastasis in the pooled cT2N0 cohort. (A) Positive predictive value [file jamasurg-e256755-s001.pdf]

Supplemental Online Content

Hong TH, Jeong J-G, Park SY, et al. Preoperative circulating tumor dna detection and risk stratification in esophageal squamous cell carcinoma. *JAMA Surg*. Published online February 18, 2026. doi:10.1001/jamasurg.2025.6755

eMethods

eFigure 1. Study workflow and patient enrollment across institutional cohorts. (A) CONSORT-style diagram depicting patient screening, exclusion, and inclusion for both institutional cohorts (SMC and YUSH). (B) Schematic representation of the study workflow

eFigure 2. Validity of cfDNA analytical parameters. (A) Alteration depth stratified by cfDNA VAF threshold of 0.1%. (B) cfDNA sequencing depth according to cfDNA input quantity (≤cutoff vs. >cutoff). (C) cfDNA amount (ng) compared between pathologic stage I and stage II–IV

eFigure 3. Model calibration plots for prediction of nodal metastasis in clinical T2N0 ESCC patients. (A) Calibration plot for the SMC cohort (n=24). (B) Calibration plot for the YUSH cohort (n=21). (C) Calibration plot for the pooled cohort (n=45).eTable 1. Clinicopathological characteristics of study population

eTable 1. Clinicopathological characteristics of study population

eFigure 4. Association between clinical stage, ctDNA detection status, and pathologic stage in ESCC patients. (A) Sankey diagram displaying the relationship between clinical stage, ctDNA detection status, and pathologic stage in the SMC cohort. (B) Sankey diagram displaying the relationship between clinical stage, ctDNA detection status, and pathologic stage in the YUSH cohort

eFigure 5. Survival outcomes in the overall ESCC cohort. (A) Kaplan-Meier curve for recurrence-free survival of all patients. (B) Kaplan-Meier curve for overall survival of all patients

eTable 2. Univariable and multivariable Cox regression analysis using preoperative risk variables for recurrence-free survival

eTable 3. Univariable and multivariable Cox regression analysis using preoperative risk variables for overall survival

eTable 4. Pathologic stage distribution by ctDNA status

eTable 5. Association between preoperative ctDNA detection and recurrence patterns

© 2026 Hong TH et al. *JAMA Surgery*.

eFigure 6. Predictive performance of ctDNA for occult nodal metastasis in the pooled cT2N0 cohort. (A) Positive predictive values (PPV) of conventional high-risk factors and ctDNA detection for pathologic nodal metastasis in the pooled cT2N0 cohort. (B) Receiver operating characteristic curves showing the added value of ctDNA incorporation to guideline-based risk assessment for predicting nodal metastasis in the pooled cT2N0 cohort

eTable 6. Diagnostic Performance Metrics of ctDNA and Conventional Risk Factors for Predicting Occult Nodal Metastasis in Clinical T2N0 ESCC Patients

eTable 7. Logistic regression analysis for prediction of nodal metastasis in the SMC cT2N0 cohort (N=24)

eTable 8. Logistic regression analysis for prediction of nodal metastasis in the YUSH cT2N0 cohort (N=21)

eTable 9. Logistic regression analysis for prediction of nodal metastasis in the combined cT1-2N0 cohort (N=53)

eTable 10. Model Calibration and Discrimination Metrics for Prediction of Nodal Metastasis

eTable 11. Net Reclassification Index Analysis

This supplemental material has been provided by the authors to give readers additional information about their work.

© 2026 Hong TH et al. *JAMA Surgery*.

Variant Calling, Error Suppression, and Copy Number Estimation

Detection of SNVs and Indels was performed using IMBdx's proprietary deepblood software, which applies background error modeling and UMI-based consensus calling to enhance specificity and sensitivity. Variants in clonal hematopoiesis of indeterminate potential (CHIP)-associated genes (ATM, CBL, CHEK2, IDH2, JAK2, MPL, U2AF1) with low variant allele frequency (VAF) or classified as variants of unknown significance (VUS) were filtered to minimize false positives. Fusion genes were identified through a three-step approach incorporating split-read and k-mer mapping, followed by stringent filtering for mapping quality and predicted functionality. Copy number alterations (CNAs) were inferred using log2 ratio comparisons to a panel of normal controls, with further validation through SNP allele frequency analysis. These procedures followed the analytical pipeline described in a prior validation study[1] which established the assay's limit of detection (LOD) at: SNVs = 0.11%, INDELs = 0.06%, fusions = 0.21%, and CNAs = 2.13 copies at 30 ng cfDNA input.

Validation of VAF Threshold and cfDNA Input Quality

To establish the analytical threshold for ctDNA positivity, we defined a VAF cutoff of 0.1%, based on the requirement of at least 10 supporting reads to ensure variant reliability at the given sequencing depth. This decision was guided by the sequencing design, where plasma cfDNA was sequenced to a median raw depth exceeding 50,000× and deduplicated depth of approximately 5,000×. At this depth, a 0.1% variant corresponds to ~5 reads; thus, variants needed to be supported by at least 10 reads for confident detection.

We validated this cutoff by analyzing sequencing metrics in the SMC cohort. Consistent with this framework, variants with VAF >0.1% typically exhibited alteration depths ≥10 ($p < 0.001$, **eFigure 2A**), supporting the analytical cutoff for reliable detection. Samples above the first quartile threshold (>Q1 ng) achieved significantly deeper deduplicated coverage than those below, with a clear separation observed around 5,000× ($p < 0.001$, **eFigure 2B**). These findings confirm the importance of sufficient cfDNA input for achieving the target sequencing depth required for 0.1% sensitivity.

Furthermore, cfDNA concentration was associated with pathological stage: patients with pathologic stage II–IV disease exhibited higher cfDNA amounts than those with stage I disease ($p = 0.013$, **eFigure 2C**), reflecting increased tumor burden and supporting the biological plausibility of our detection framework.

References

1. Yi H, Youk J, Lim Y, et al: Analytical and Clinical Validation of a Highly Sensitive NGS-Based ctDNA Assay with Real-World Concordance in Non–Small Cell Lung Cancer. Cancer Res Treat 56:765-773, 2024

© 2026 Hong TH et al. *JAMA Surgery*.

eFigure 1. Study workflow and patient enrollment across institutional cohorts.

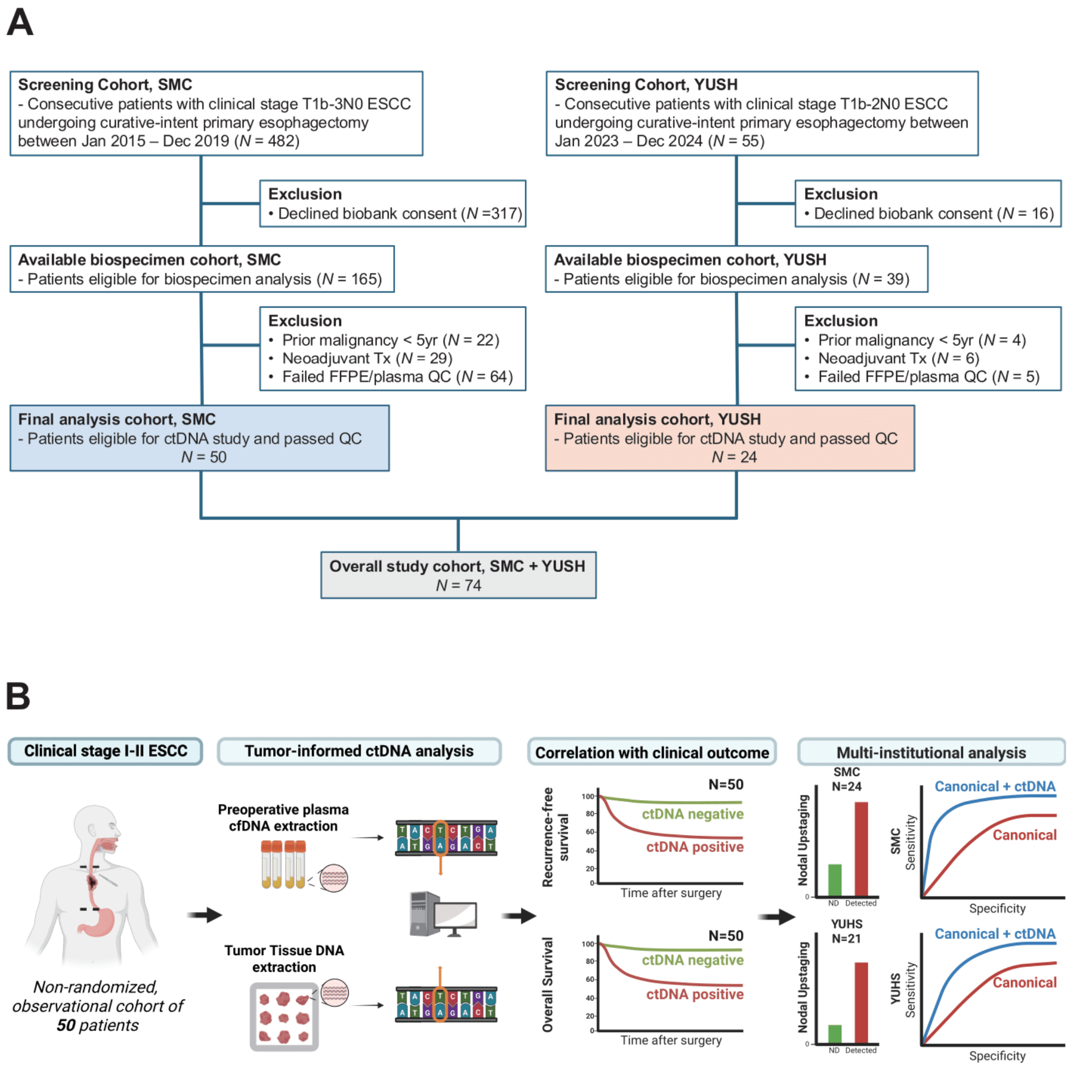

© 2026 Hong TH et al. *JAMA Surgery*.

eFigure 2. Validity of cfDNA analytical parameters.

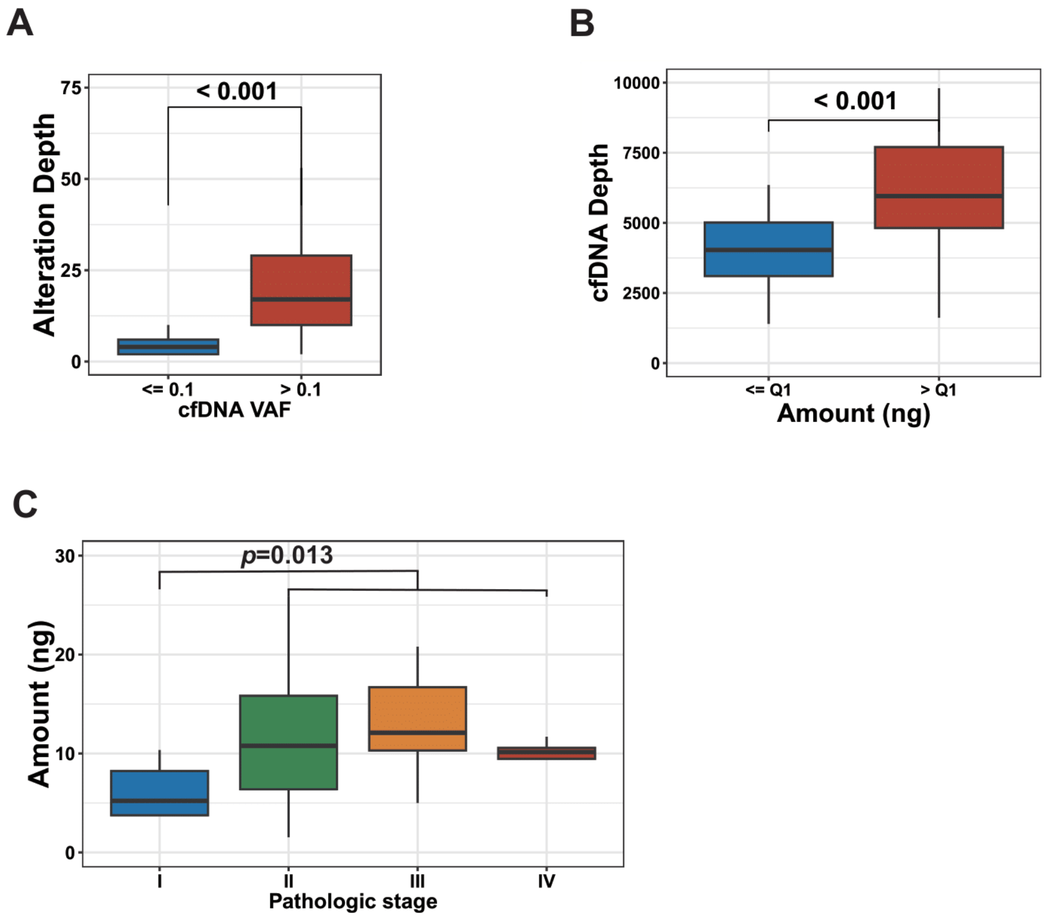

© 2026 Hong TH et al. *JAMA Surgery*.

eFigure 3. Model calibration plots for prediction of nodal metastasis in clinical T2N0 ESCC patients.

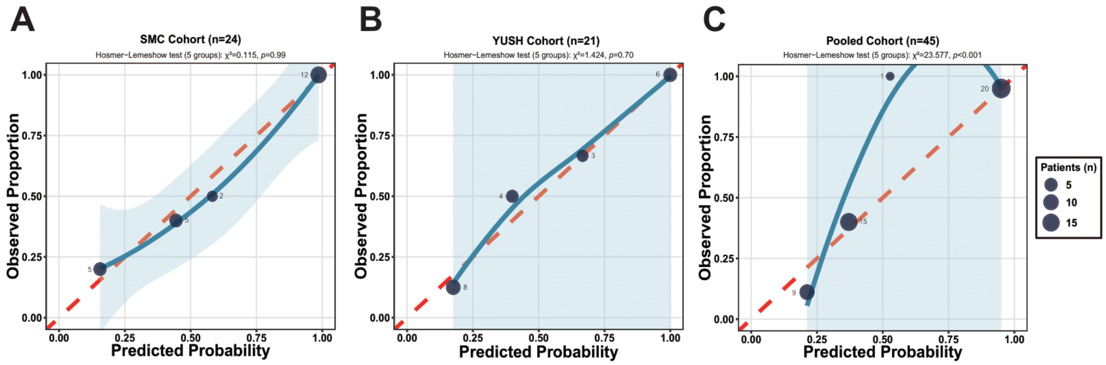

© 2026 Hong TH et al. *JAMA Surgery*.

eTable 1. Clinicopathological characteristics of study population

Characteristics	SMC cohort (N = 50)	YUSH cohort (N = 24)
Age	68 [60, 74]	67 [61, 69]
Sex		
Female	3 (6.0%)	3 (12.5%)
Male	47 (94%)	21 (87.5%)
Smoker	40 (80%)	20 (83.3 %)
Location		
Upper	7 (14%)	4 (16.7%)
Middle	19 (38%)	10 (41.7%)
Lower	24 (48%)	10 (41.7%)
Operation type		
Ivor Lewis operation	38 (76%)	1 (4.2%)
McKeown operation	12 (24%)	23 (95.8%)
Surgical approach		
RATS	19 (38%)	22 (91.6%)
VATS	7 (14%)	1 (4.2%)
Open	24 (48%)	1 (4.2%)
Extent of LND		
2-Field	43 (86%)	1 (4.2%)
3-Field	7 (14%)	23 (95.8%)
Preoperative PET-CT		
Performed	50 (100%)	24 (100%)
Preoperative EUS		
Performed	50 (100%)	24 (100%)
Clinical T stage		
T1b	5 (10%)	3 (12.5%)
T2	24 (48%)	21 (87.5%)
T3	21 (42%)	0 (0%)
Clinical N stage		

© 2026 Hong TH et al. *JAMA Surgery*.

N0	50 (100%)	24 (100%)
Clinical stage		
I	5 (10%)	3 (12.5%)
II	45 (90%)	21 (87.5%)
The number of LN dissected[†]	43 (17 - 76)	58 (42 - 89)

[1]n (%) or median [IQR]; [†]LN count presented as median (range)

EUS, endoscopic ultrasound; LND, lymph node dissection; PET-CT, positron emission tomography–computed tomography; RATS, robotic-assisted thoracoscopic surgery; VATS, video-assisted thoracoscopic surgery.

© 2026 Hong TH et al. *JAMA Surgery*.

eFigure 4. Association between clinical stage, ctDNA detection status, and pathologic stage in ESCC patients.

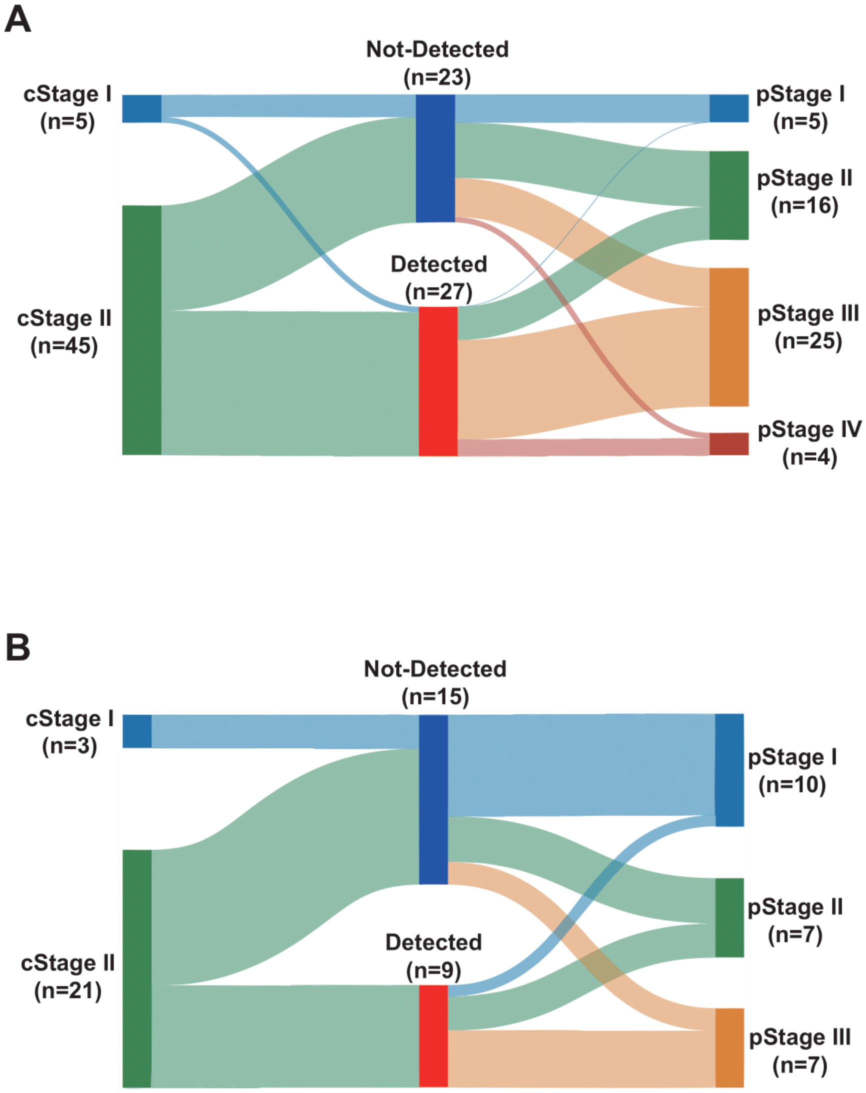

© 2026 Hong TH et al. *JAMA Surgery*.

eFigure 5. Survival outcomes in the overall ESCC cohort.

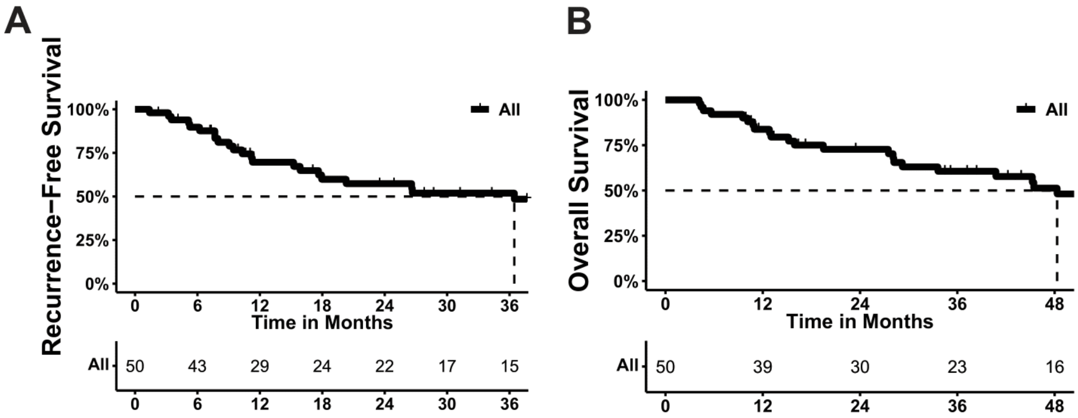

© 2026 Hong TH et al. *JAMA Surgery*.

eTable 2. Univariable and multivariable Cox regression analysis using preoperative risk variables for recurrence-free survival

	**Univariable**			**Multivariable**		
Characteristic	Crude HR	95% CI	*p*	Adjusted HR	95% CI	*p*
Age	0.99	(0.943-1.05)	0.871	0.98	(0.92-1.05)	0.567
cT						
cT1	-	-	-	-	-	-
cT2-3	3.67	(0.49-27.31)	0.204	1.49	(0.16-14.14)	0.726
Tumor Size						
< 3 cm	-	-	-	-	-	-
≥ 3 cm	5.22	(0.70-38.78)	0.106	2.61	(0.28-24.24)	0.399
LVI						
Negative	-	-	-	-	-	-
Positive	1.4	(0.62-3.18)	0.420	1.11	(0.47-2.61)	0.820
Grade						
G1-2	-	-	-	-	-	-
G3	0.92	(0.31-2.73)	0.884	1.56	(0.46-5.26)	0.475
**ctDNA**						
Not Detected	-	-	-	-	-	-
**Detected**	**4.15**	**(1.54-11.22)**	**0.00504**	**4.07**	**(1.27-13.01)**	**0.0179**

CI, Confidence Interval; ctDNA, circulating-tumor DNA; HR, Hazard Ratio; LVI, Lymphovascular Invasion.

© 2026 Hong TH et al. *JAMA Surgery*.

eTable 3. Univariable and multivariable Cox regression analysis using preoperative risk variables for overall survival

	**Univariable**			**Multivariable**		
Characteristic	Crude HR	95% CI	*p*	Adjusted HR	95% CI	*p*
Age	1.03	(0.97-1.09)	0.299	1.02	(0.96-1.08)	0.591
cT						
cT1	-	-	-	-	-	-
cT2-3	2.36	(0.54-10.33)	0.253	1.28	(0.16-10.45)	0.8201
Tumor Size						
< 3 cm	-	-	-	-	-	-
≥ 3 cm	2.75	(0.64-11.89)	0.175	2.44	(0.29-20.21)	0.408
LVI						
Negative	-	-	-	-	-	-
Positive	1.01	(0.44-2.30)	0.986	0.95	(0.41-2.25)	0.915
Grade						
G1-2	-	-	-	-	-	-
G3	0.71	(0.21-2.38)	0.575	0.92	(0.26-3.32)	0.9002
**ctDNA**						
Not Detected	-	-	-	-	-	-
**Detected**	**4.02**	**(1.50-10.74)**	**0.00554**	**3.54**	**(1.24-10.05)**	**0.0178**

CI, Confidence Interval; ctDNA, circulating-tumor DNA; HR, Hazard Ratio; LVI, Lymphovascular Invasion.

© 2026 Hong TH et al. *JAMA Surgery*.

eTable 4. Pathologic stage distribution by ctDNA status.

**Characteristics**	**ctDNA-negative (N=38)**	**ctDNA-positive (N=36)**	***p***
Pathologic T stage			<0.001
T1	18 (47.4%)	6 (16.7%)	
T2	10 (26.3%)	5 (13.9%)	
T3	10 (26.3%)	25 (69.4%)	
Pathologic N Stage			<0.001
N0	24 (63.2%)	6 (16.7%)	
N1	10 (26.3%)	11 (30.5%)	
N2-3	4 (10.5%)	19 (52.8%)	

© 2026 Hong TH et al. *JAMA Surgery*.

eTable 5. Association between preoperative ctDNA detection and recurrence patterns

	**ctDNA-negative**	**ctDNA-positive**	*p*
**Not Recurred**	18 (78.3%)	9 (33.3%)	0.002
**Recurred**	5 (21.7%)	18 (66.7%)	
Locoregional	2 (8.7%)	5 (18.5%)	0.430
- mLN	1 (4.3%)	5 (18.5%)	
- Anastomosis site	1 (4.3%)	0 (0.0%)	
Systemic	3 (13%)	13 (48.1%)	0.014
- Lung	2 (8.6%)	3 (11.1%)	
- Liver	1 (4.3%)	2 (7.4%)	
- Brain		2 (7.4%)	
- Bone		2 (7.4%)	
- Peritoneal seeding		1 (3.7%)	
- Pancreas		1 (3.7%)	
- Distant lymph nodes		2 (7.4%)	

mLN, mediastinal lymph node; ctDNA, circulating-tumor DNA.

eFigure 6. Predictive performance of ctDNA for occult nodal metastasis in the pooled cT2N0 cohort. (A) Positive predictive values (PPV) of conventional high-risk factors and ctDNA detection for pathologic nodal metastasis in the pooled cT2N0 cohort. (B) Receiver operating characteristic curves showing the added value of ctDNA incorporation to guideline-based risk assessment for predicting nodal metastasis in the pooled cT2N0 cohort.

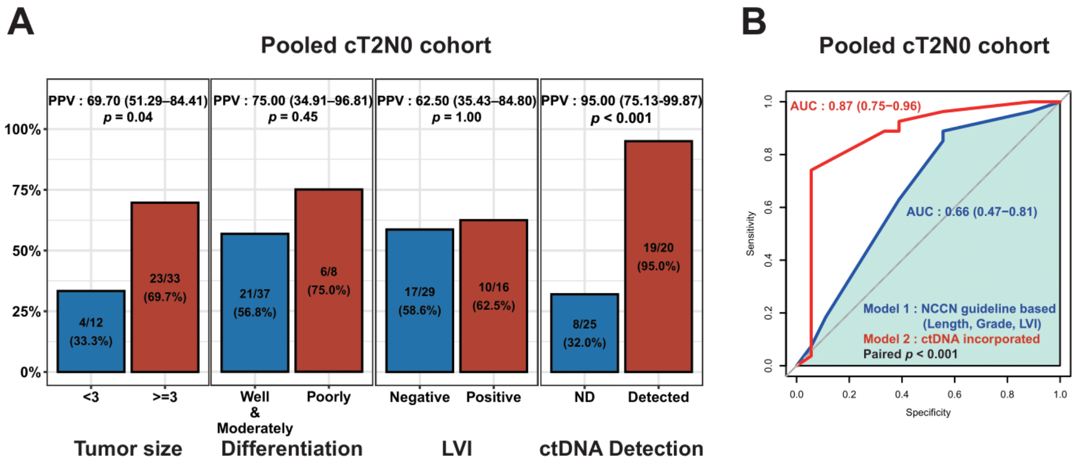

© 2026 Hong TH et al. *JAMA Surgery*.

eTable 6. Diagnostic Performance Metrics of ctDNA and Conventional Risk Factors for Predicting Occult Nodal Metastasis in Clinical T2N0 ESCC Patients.

**Pooled**	**Sensitivity**	**Specificity**	**PPV**	**NPV**
**Tumor size**	85.19% (66.27–95.81%)	44.44% (21.53–69.24%)	69.70% (51.29–84.41%)	66.67% (34.89–90.08%)
**Differentiation**	22.22% (8.62–42.26%)	88.89% (65.29–98.62%)	75.00% (34.91–96.81%)	43.24% (27.10–60.51%)
**LVI**	37.04% (19.40–57.63%)	66.67% (40.99–86.66%)	62.50% (35.43–84.80%)	41.38% (23.52–61.06%)
**ctDNA**	70.37% (49.82–86.25%)	94.44% (72.71–99.86%)	95.00% (75.13–99.87%)	68.00% (46.50–85.05%)
**SMC**	**Sensitivity**	**Specificity**	**PPV**	**NPV**
**Tumor size**	87.50% (61.65–98.45%)	37.50% (8.52–75.51%)	73.68% (48.80–90.85%)	60.00% (14.66–94.73%)
**Differentiation**	25.00% (7.27–52.38%)	87.50% (47.35–99.68%)	80.00% (28.36–99.49%)	36.84% (16.29–61.64%)
**LVI**	43.80% (19.75–70.12%)	62.50% (24.49–91.48%)	70.00% (34.75–93.33%)	35.71% (12.76–64.86%)
**ctDNA**	68.75% (41.34–88.98%)	100.00% (63.06–100.0%)	100.00% (71.51–100.0%)	61.54% (31.58–86.14%)
**YUHS**	**Sensitivity**	**Specificity**	**PPV**	**NPV**
**Tumor size**	81.82% (48.22–97.72%)	50.00% (18.71–81.29%)	64.29% (35.14–87.24%)	71.43% (29.04–96.33%)
**Differentiation**	18.18% (2.28–51.78%)	90.00% (55.50–99.75%)	66.67% (9.43–99.16%)	50.00% (26.02–73.98%)
**LVI**	27.27% (6.02–60.97%)	70.00% (34.75–93.33%)	50.00% (11.81–88.19%)	46.67% (21.27–73.41%)
**ctDNA**	72.73% (39.03–93.98%)	90.00% (55.50–99.75%)	88.89% (51.75–99.72%)	75.00% (42.81–94.51%)

© 2026 Hong TH et al. *JAMA Surgery*.

eTable 7. Logistic regression analysis for prediction of occult nodal metastasis in the SMC cT2N0 cohort (N=24)

	Univariable			Multivariable		
**Characteristic**	**OR[1]**	**95% CI**	***p***	**OR[1]**	**95% CI**	***p***
**Tumor size**						
<3cm	-	-		-	-	
≥ 3cm	4.20	0.55, 40.04	0.17	2.22	0.24, 31.63	0.49
**Grade**						
G1-2	-	-		-	-	
G3	2.33	0.27, 50.63	0.49	3.39	0.34, 48.66	0.30
**LVI**						
Negative	-	-		-	-	
Positive	1.30	0.23, 8.15	0.77	0.46	0.03, 4.10	0.49
**ctDNA**						
Not detected	-	-		-	-	
Detected	35.55	3.37, 4918.09	0.0011	33.96	2.98, 4994.37	0.0021

OR, Odds Ratio; CI, Confidence Interval; ctDNA, circulating-tumor DNA.

© 2026 Hong TH et al. *JAMA Surgery*.

eTable 8. Logistic regression analysis for prediction of occult nodal metastasis in the YUSH cT2N0 cohort (N=21)

	Univariable			Multivariable		
**Characteristic**	**OR[1]**	**95% CI**	***p***	**OR[1]**	**95% CI**	***p***
**Tumor size**						
<3cm	-	-		-	-	
≥ 3cm	4.50	0.69, 40.70	0.13	2.29	0.22, 31.47	0.49
**Grade**						
G1-2	-	-		-	-	
G3	2.00	0.16, 47.71	0.60	0.13	0.00, 3.15	0.21
**LVI**						
Negative	-	-		-	-	
Positive	0.88	0.12, 6.15	0.89	0.54	0.04, 4.97	0.60
**ctDNA**						
Not detected	-	-		-	-	
Detected	15.38	2.25, 190.43	0.0041	22.43	2.01, 3048.69	0.0086

OR, Odds Ratio; CI, Confidence Interval; ctDNA, circulating-tumor DNA.

© 2026 Hong TH et al. *JAMA Surgery*.

eTable 9. Logistic regression analysis for prediction of nodal metastasis in the combined cT1-2N0 cohort (N=53)

	**Univariable**			**Multivariable**		
**Characteristic**	**OR[1]**	**95% CI**	***p***	**OR[1]**	**95% CI**	***p***
**Tumor size**						
<3cm	-	-		-	-	
≥ 3cm	2.68	0.81, 9.25	0.11	1.49	0.38, 6.05	0.56
**Grade**						
G1-2	-	-		-	-	
G3	1.19	0.33, 5.13	0.80	0.92	0.18, 4.57	0.92
**LVI**						
Negative	-	-		-	-	
Positive	1.05	0.33, 3.46	0.94	0.59	0.13, 2.42	0.47
**ctDNA**						
Not detected	-	-		-	-	
Detected	22.41	4.75, 219.88	< 0.001	19.11	4.01, 189.44	<0.001

OR, Odds Ratio; CI, Confidence Interval; ctDNA, circulating-tumor DNA.

© 2026 Hong TH et al. *JAMA Surgery*.

eTable 10. Model Calibration and Discrimination Metrics for Prediction of Nodal Metastasis.

Metric	Pooled Cohort (n=45)	SMC Cohort (n=24)	YUSH Cohort (n=21)
Hosmer-Lemeshow test			
χ^2	23.577	0.115	1.424
Degrees of freedom	3	3	3
P-value	<0.001	0.99	0.7
Calibration Metrics†			
Slope	1	1	1
Intercept	0	0	0
Predictive Performance			
Brier score‡	0.128	0.105	0.132
C-statistic (AUC) §	0.872	0.91	0.886

† Calculated by regressing observed outcomes on predicted log-odds

‡ Mean squared difference between predicted probabilities and observed outcomes; values <0.25 indicate acceptable performance

§ Area under the receiver operating characteristic curve

© 2026 Hong TH et al. *JAMA Surgery*.

eTable 11. Net Reclassification Index Analysis.

Cohort	N	Categorical NRI (95% CI)	P-value	Continuous NRI (95% CI)	P-value	IDI (95% CI)	P-value
Pooled	45	1.111 (0.773-1.449)	<0.001	1.296 (0.892-1.701)	<0.001	0.334 (0.210-0.457)	<0.001
SMC	24	0.938 (0.497-1.378)	<0.001	1.500 (1.076-1.924)	<0.001	0.416 (0.252-0.580)	<0.001
YUSH	21	1.055 (0.562-1.547)	<0.001	1.073 (0.393-1.752)	0.002	0.349 (0.140-0.557)	0.001

© 2026 Hong TH et al. *JAMA Surgery*.
