# Supplementary material for: Preoperative Circulating Tumor DNA Detection and Risk Stratification in Esophageal Squamous Cell Carcinoma
Source: JAMA Surg. 2026 Feb 18;161(4):370–9. doi: 10.1001/jamasurg.2025.6755 (PMC12917745; doi:10.1001/jamasurg.2025.6755)
Supplement: Supplement 2. — Data sharing statement [file jamasurg-e256755-s002.pdf]

Data Sharing Statement

Hong. Preoperative Circulating Tumor DNA Detection and Risk Stratification in Esophageal Squamous Cell Carcinoma. *JAMA Surg*. Published February 18, 2026.
doi:10.1001/jamasurg.2025.6755

Data
Data available: Yes
Data types: Deidentified participant data
How to access data: hkts@skku.edu
When available: With publication

Supporting Documents
Document types: Statistical/analytic code
How to access documents: hkts@skku.edu
When available: With publication

Additional Information
Who can access the data: researchers whose proposed use of the data has been approved
Types of analyses: research-use only
Mechanisms of data availability: with a signed data access agreement
